# Supplementary figures and images for: Guanidine thiocyanate solution facilitates sample collection for plant rhizosphere microbiome analysis
Source: PeerJ. 2019 Feb 19;7:e6440. doi: 10.7717/peerj.6440 (PMC6385689; doi:10.7717/peerj.6440)

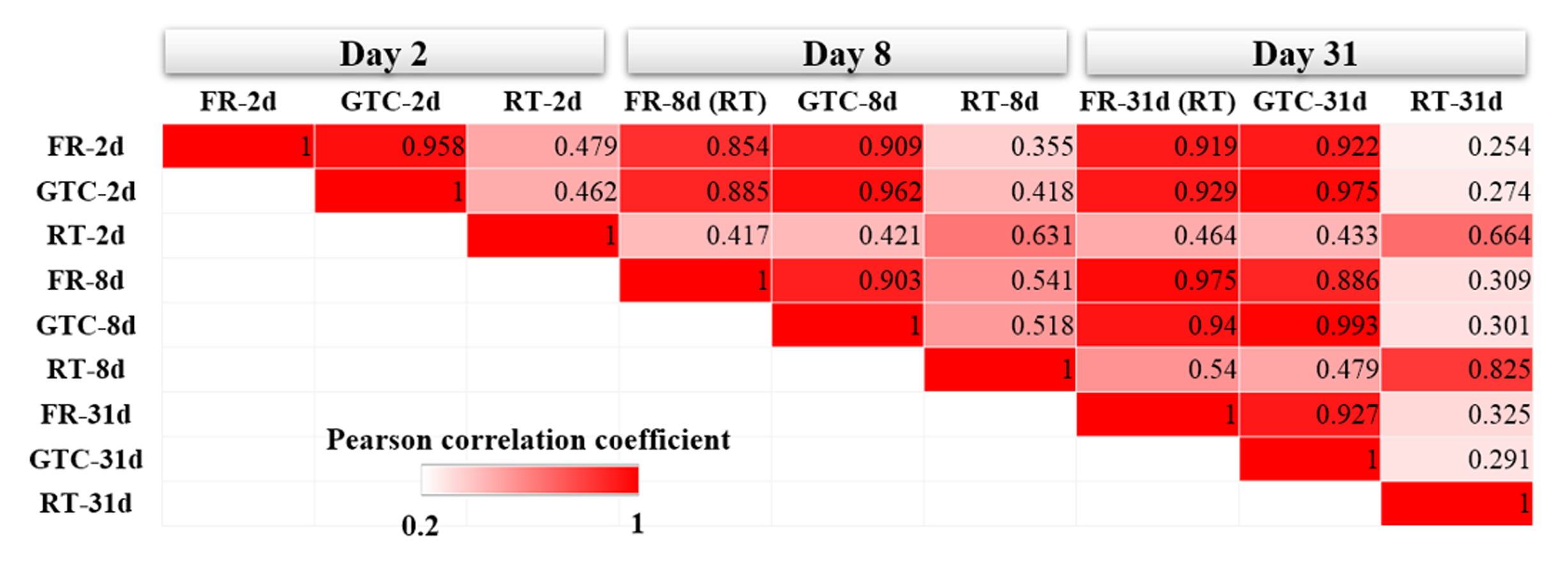

Supplement: Supplemental Information 3 — FR-2d, rhizosphere samples stored at −7 °C for 2 days (n = 3). FR-8d (RT) and FR-31d (RT) are rhizosphere samples stored at −7 °C for 8 and 31 days, respectively, thawed at room temperature for 15 min before DNA isolation (n = 3). GTC-2d, -8d, and 31d are samples stored in 100 μL of GTC guanidine thiocyanate at room temperature for 24 h, and −20 ° for 1, 7, and 30 days, respectively (n = 3). RT-2d, -8d, and 31d are samples stored at room temperature for 2, 8, and 31 days, respectively (n = 3). [file peerj-07-6440-s003.png]
